# Modeling HIV-HCV coinfection epidemiology in the direct-acting antiviral era: the road to elimination

**DOI:** 10.1186/s12916-017-0979-1

**Published:** 2017-12-18

**Authors:** Victor Virlogeux, Fabien Zoulim, Pascal Pugliese, Isabelle Poizot-Martin, Marc-Antoine Valantin, Lise Cuzin, Jacques Reynes, Eric Billaud, Thomas Huleux, Firouze Bani-Sadr, David Rey, Anne Frésard, Christine Jacomet, Claudine Duvivier, Antoine Cheret, Laurent Hustache-Mathieu, Bruno Hoen, André Cabié, Laurent Cotte, the Dat’AIDS Study Group

**Affiliations:** 1 Department of Hepatology, Croix-Rousse Hospital, Hospices Civils de Lyon, Lyon, France; 2 Université Claude Bernard Lyon 1, INSERM 1052, CNRS 5286, Centre Léon Bérard, Centre de Recherche en Cancérologie de Lyon, F-69008 Lyon, France; 3 Centre for Clinical Research, Department of Hepatology, Groupement Hospitalier Nord, Hospices Civils de Lyon, Lyon, France; 4 Department of Infectious Diseases, Centre Hospitalier Universitaire de Nice, Hôpital l’Archet, Nice, France; 5 Aix-Marseille University, APHM Hôpital Sainte-Marguerite, Service d’Immuno-hématologie clinique, INSERM U912 (SESSTIM), 13009 Marseille, France; 6 Department of Infectious Diseases, Assistance Publique - Hôpitaux de Paris, Pitié-Salpêtrière Hospital, Paris, France; 7 Sorbonne Université, UPMC Université Paris 06, INSERM, Institut Pierre Louis d’Epidémiologie et de Santé Publique (IPLESP UMRS 1136), Paris, France; 8 CHU Toulouse, COREVIH Toulouse, Toulouse, France; 9 Université de Toulouse III, Toulouse, France; 10 INSERM, UMR 1027, Toulouse, France; 11 Department of Infectious Diseases, UMI 233 INSERM U1175, CHU de Montpellier, Montpellier, France; 12 Department of Infectious Diseases, Hotel-Dieu Hospital, Nantes, France; 13 Department of Infectious Diseases and Travel Diseases, Centre Hospitalier Gustave-Dron, Tourcoing, France; 14 Department of Internal Medicine, Infectious Diseases and Clinical Immunology, Hôpital Robert Debré, CHU Reims, Reims, France; 15 Université de Reims Champagne-Ardenne, Faculté de Médecine, EA-4684/SFR CAP-SANTE, Reims, France; 16 HIV Infection Care Centre, Hôpitaux Universitaires, Strasbourg, France; 17 Department of Infectious Diseases, CHU Saint-Etienne, Saint-Priest-en-Jarez, France; 18 Department of Infectious Diseases, CHU Clermont-Ferrand, Clermont-Ferrand, France; 19 Department of Infectious Diseases, Centre d’Infectiologie Necker-Pasteur, IHU Imagine, Assistance Publique - Hôpitaux de Paris, Hôpital Necker-Enfants Malades, Paris, France; 20 Université Paris Descartes, Sorbonne Paris Cité, EA7327, Paris, France; 21 Department of Internal Medicine, CHU Bicètre, Paris, France; 22 Department of Infectious Diseases, CHRU de Besançon, Besançon, France; 23 Faculté de Médecine Hyacinthe Bastaraud, Université des Antilles, and Service de Maladies Infectieuses et Tropicales, Dermatologie et Médecine Interne, and INSERM CIC 1424, Centre Hospitalier Universitaire de Pointe-à-Pitre, Pointe-à-Pitre, France; 24 Department of Infectious Diseases, CHU de Martinique, Fort-de-France, France; 25 Université des Antilles EA4537 and INSERM CIC1424, Fort-de-France, France; 26 Department of Infectious Diseases and Tropical Medicine, Croix-Rousse Hospital, Hospices Civils de Lyon, 103 grande rue de la Croix-Rousse, 69317 Lyon, CEDEX 04 France; 27 Lyon University, Lyon, France

**Keywords:** HIV, HCV, Coinfection, Treatment uptake, Mathematical modeling, Compartmental model, Direct-acting antiviral agent, HCV elimination

## Abstract

**Background:**

HCV treatment uptake has drastically increased in HIV-HCV coinfected patients in France since direct-acting antiviral (DAA) treatment approval, resulting in HCV cure in 63% of all HIV-HCV patients by the end of 2015. We investigated the impact of scaling-up DAA on HCV prevalence in the whole HIV population and in various risk groups over the next 10 years in France using a transmission dynamic compartmental model.

**Methods:**

The model was based on epidemiological data from the French Dat’AIDS cohort. Eight risk groups were considered, including high-risk (HR) and low-risk (LR) men who have sex with men (MSM) and male/female heterosexuals, intra-venous drug users, or patients from other risk groups. The model was calibrated on prevalence and incidence data observed in the cohort between 2012 and 2015.

**Results:**

On January 1, 2016, 156,811 patients were registered as infected with HIV in France (24,900 undiagnosed patients) of whom 7938 (5.1%) had detectable HCV-RNA (722 undiagnosed patients). Assuming a treatment coverage (TC) rate of 30%/year (i.e., the observed rate in 2015), model projections showed that HCV prevalence among HIV patients is expected to drop to 0.81% in 2026. Sub-analyses showed a similar decrease of HIV-HCV prevalence in most risk groups, including LR MSM. Due to higher infection and reinfection rates, predicted prevalence in HR MSM remained stable from 6.96% in 2016 to 6.34% in 2026. Increasing annual TC rate in HR MSM to 50/70% would decrease HCV prevalence in this group to 2.35/1.25% in 2026. With a 30% TC rate, undiagnosed patients would account for 34% of HCV infections in 2026.

**Conclusions:**

Our model suggests that DAA could nearly eliminate coinfection in France within 10 years for most risk groups, including LR MSM. Elimination in HR MSM will require increased TC.

**Electronic supplementary material:**

The online version of this article (doi:10.1186/s12916-017-0979-1) contains supplementary material, which is available to authorized users.

## Background

Chronic hepatitis C virus (HCV) infection affects approximately 100 million people worldwide [1]. HCV coinfection is common among patients infected by human immunodeficiency virus (HIV), with a prevalence of 15.1% in 2012 in France [2]. HIV-HCV coinfection leads to accelerated disease progression, i.e., accelerated hepatic fibrosis progression and higher rates of liver decompensation and death [3, 4]. International guidelines consequently recommend that coinfected patients should be given high priority for HCV treatment, regardless of the fibrosis stage [5, 6].

HCV direct-acting antiviral (DAA) agents are associated with a sustained virological response (SVR) rate of over 90% regardless of the genotype, including in HIV-infected patients [7, 8]. However, recent studies reported that, among HIV-infected men who have sex with men (MSM), HCV reinfection rates are alarmingly high and that specific strategies, such as frequent HCV RNA testing, were more particularly needed for this risk group [9–12]. Of note, such alarming HCV reinfection incidence rates have not been reported thus far for other risk groups. Recent modeling studies focusing on the MSM population showed that, despite high DAA treatment rates, the HCV epidemic would continue unless more effective behavioral interventions were undertaken in this population [13, 14].

To explore the potential impact of DAA treatment on HIV-HCV epidemic in France, we used incidence and prevalence data from a large HIV multicentric cohort [2, 15]. We developed a dynamic compartmental model of HCV transmission to assess the impact over the next 10 years of scaled-up HCV treatment across different risk groups.

## Methods

### Epidemiological data

The Dat’AIDS cohort is a collaborative network of 15 French HIV treatment centers covering approximately 25% of HIV-infected patients followed in France (Clinicaltrials.gov ref NCT02898987) [15]. HCV incidence and prevalence data, and HCV treatment coverage and SVR rates have been collected yearly within this cohort from January 2000 onwards [2]. Patients were divided into eight different risk groups, namely males and females for heterosexuals, intra-venous drug users (IVDU) and other risk-groups, and low- and high-risk for MSM. Death rates, proportion of HCV spontaneous clearance, and SVR rates were determined from the Dat’AIDS cohort [16]. HCV reinfection was defined as a positive HCV-RNA for more than 6 months following the end of a successful HCV treatment or following a spontaneous clearance, or HCV infection with a different genotype, regardless of the time period [2].

### Estimation of the proportion of low- and high-risk MSM

As observed in other cohort studies [9–12], HCV reinfection incidence in MSM was higher than first infection incidence in our cohort. We therefore assumed a heterogeneous risk of HCV infection among MSM [17]. We considered that the observed reinfection rate in our cohort was representative of a first infection rate in a subgroup of MSM with high-risk behaviors, as it is unlikely that, after a first HCV infection, MSM increased their risk for HCV reinfection. On the other hand, we considered that MSM with low-risk behaviors had a similar first infection rate as other risk groups and we estimated it using the mean first infection incidence observed in other risk groups each calendar year. We thus estimated the proportion of high- and low-risk HIV-monoinfected MSM according to these assumptions (Additional file 1: Appendix).

### Extrapolation of the Dat'AIDS cohort data to the total number of HIV patients in France

Prevalence of coinfection in each risk group was extrapolated from the Dat'AIDS cohort to the total number of HIV patients under care each year in France [18]. The extrapolated numbers in each risk group of HIV-monoinfected patients, HIV-HCV coinfected patients with active HCV infection (defined as detectable plasma HCV-RNA), and patients with SVR following DAA treatment or with HCV spontaneous clearance on January 1, 2016, are described in Additional file 1: Tables S1, S2, and S3 [16].

### Estimation of the HIV undiagnosed population

Considering that the HIV undiagnosed population [19] could significantly fuel the epidemic in the future, we estimated the number of HIV-HCV coinfected patients in this population and included it in the model projections (Additional file 1: Table S4). To estimate the number of coinfected patients in the undiagnosed HIV population, we assumed that HCV prevalence in this population was similar to the observed HCV prevalence among patients that were included in the Dat’AIDS cohort in 2015 with a mean of 2.9%. We therefore estimated the number of coinfected patients in each subgroup using the estimates of the overall HIV undiagnosed population divided into subgroups reported by Supervie et al. [19]. These patients were considered as non-eligible for HCV DAA treatment and the population size was assumed to be constant over time.

### Mathematical model

**Fig. 1:**
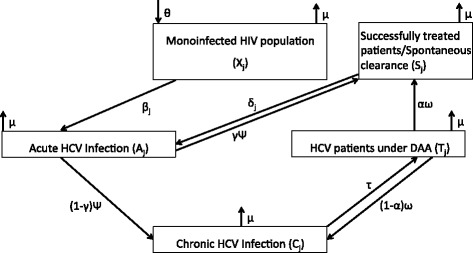
Schematic diagram of HCV transmission compartmental model. Individuals are distributed in eight different risk groups (*j*): males and females for heterosexuals, for intravenous drug users, and for other groups, and low- and high-risk subgroups for men who have sex with men. New individuals enter the susceptible monoinfected categories (X) at a rate θ [20]. Susceptible individuals may be acutely infected (A) at an infection rate *β*_*j*_ (estimated during the calibration process for each subgroup). Individuals acutely infected may progress to HCV chronic infection (C) at a rate (1–*γ*) or spontaneously clear their infection at a rate *γ* (S). Acute infection lasts an average *1*/*ψ*. Chronically infected individuals start an HCV treatment at an annual rate *τ*. Treatment lasts an average *1*/*ω* and results in SVR12 in a proportion *α* of all treated patients. Successfully treated patients or patients with spontaneous clearance may be reinfected with an external force of infection *δ*_*j*_

We developed a dynamic and deterministic model of HCV transmission, progression, and treatment among the whole under-care HIV population in France described in Fig. 1. Patients enter the model at HIV diagnosis time. The number of new HIV infections arriving in the compartment of monoinfected HIV patients (X_j_) each year was estimated using the French National Registries [20, 21]. For each compartment, the model is stratified into eight risk groups as previously described. HCV disease progression is stratified into acute phase, chronic phase, treatment phase, and successfully treated (SVR) phase. We assumed that HIV-infected patients are infecting each other within each subgroup (heterosexual, MSM, IVDU, and others) for the first infection with a proportional mixing hypothesis. Regarding reinfection, we considered a constant risk derived from observed reinfection incidence. Since acute HCV infections are rarely reported among non-HIV infected MSM, we did not consider any external force of infection in the model for this population. Projection of HIV-HCV coinfection started from January 1, 2016. Different annual treatment coverage rates were considered for projections (Additional file 1: Appendix).

#### Parameters and calibration of the model

The model was calibrated on the first infection rate β using yearly incidence and prevalence data (raw numbers) observed within the cohort from January 2012 to January 2016 and a Poisson-based likelihood within a Bayesian framework with a Monte Carlo Markov Chain method (Table 1, Additional file 1: Table S5 and Additional file 1: Appendix). We fitted our model (1) to the prevalence data observed in the Dat'AIDS cohort and extrapolated to the whole under-care HIV population in France for each risk group on January 1 between 2012 and 2016; and (2) to the incidence data observed in the Dat'AIDS cohort, i.e., the number of new first HCV infections in each risk group extrapolated to the total number of HIV under-care patients in France between 2012 and 2016. Specific HCV treatment and SVR rates were considered each year during the calibration period using observed data. Most parameters were measured from the Dat’AIDS cohort and are reported in Table 2. All HIV-HCV patients were considered eligible for HCV treatment regardless of fibrosis stage or genotype and if they were in the chronic phase of infection as defined by a detectable HCV-RNA more than 6 months following acute infection.

**Table 1:** Annual first infection rate, reinfection rate, and HCV treatment coverage observed in the Dat'AIDs cohort between 2012 and 2016

	MSM (low-risk/high-risk)	Heterosexuals	IVDU	Others
2012				
First infection rate (per 100 py)	0.02/1.84	0.01	0	0.09
Reinfection rate (per 100 py)	0.22^a^/2.56^c^	0.24^b^	0.16^b^	0.27^b^
HCV treatment rate (%)	13.4	6.9	6.8	10.9
2013				
First infection rate (per 100 py)	0.06/2.23	0.03	0	0.29
Reinfection rate (per 100 py)	0.22^a^/2.56^c^	0.24^b^	0.16^b^	0.27^b^
HCV treatment rate (%)	4.4	11.0	5.1	7.6
2014				
First infection rate (per 100 py)	0.12/2.40	0.07	2.13	0.23
Reinfection rate (per 100 py)	0.22^a^/2.56^c^	0.24^b^	0.16^b^	0.27^b^
HCV treatment rate (%)	10.5	14.0	17.1	23.0
2015				
First infection rate (per 100 py)	0.09/3.42	0.05	1.69	0.17
Reinfection rate (per 100 py)	0.22^a^/2.56^c^	0.24^b^	0.16^b^	0.27^b^
HCV treatment rate (%)	27.6	36.0	27.0	33.8

^a^Mean yearly reinfection rate observed in non-MSM risk groups between 2012 and 2015

^b^Mean yearly reinfection rate observed in heterosexuals, IVDU, and others risk groups between 2012 and 2015

^c^Mean yearly reinfection rate observed in MSM between 2012 and 2015

*HCV* hepatitis C virus, *IVDU* intravenous drug users, *MSM* men who have sex with men, *py* person-year

**Table 2:** Model parameters used to fit the incidence/prevalence of HCV patients observed in the Dat’AIDS cohort between 2012 and 2016 and for the different projections

Parameter	Symbol	Point value	Unit	References
Death rate	μ	1.4%	per year	Dat’AIDS
Infection rate of HIV (in each risk group *j*)	θ_j_	see Reference	–	[19, 20]
Proportion of HCV spontaneous clearance	γ	12.6%	–	Dat’AIDS
Mean duration of acute infection	1/Ψ	180	days	[9]
Infection rate of HCV among HIV patients in each risk group *j*	β_j_	estimated	–	Dat’AIDS
Treatment coverage rate	τ	(30–90)	per year	–
Average treatment duration	1/ω	12 (since 2015) and 24 (before 2015)	weeks	–
DAA treatment SVR12 rate	α	95%	–	Dat’AIDS
Re-infection rate of HCV among HIV patients after SVR12 or spontaneous clearance in each risk group *j*	δ_j_	see Table 1	per year	Dat’AIDS

*DAA* direct-acting antivirals, *HCV* hepatitis C virus, *HIV* human immunodeficiency virus, *SVR* sustained virological response

#### Sensitivity analysis

To estimate the potential impact of targeting HIV-HCV coinfected high-risk MSM for treatment during the HCV acute phase, we derived a model considering potential DAA treatment during this phase (i.e., from 3 to 6 months following infection) with different coverage rates (Additional file 2: Figure S1) [5, 6]. We also explored the impact of potential behavioral changes over the next 10 years among high-risk MSM on HIV-HCV prevalence by considering a linear increase in the proportion of HIV monoinfected high-risk MSM.

We also investigated, as a sensitivity analysis, the impact of considering a potential external source of HCV transmission for IVDU, i.e., from HIV-negative to HIV-positive IVDU, on the model projections over the next 10 years. We derived the main model by adding a constant external force of infection (i.e., independent of HCV prevalence among HIV-positive IVDU) among IVDU. To estimate this external force of infection, we considered several rates (20%, 40%, 60%, 80%, and 100%) of potentially observed external HCV cases among IVDU in the Dat'AIDS cohort. We therefore calibrated our model again on the adjusted dataset to take into account both internal and external forces of infection during the calibration process and considered two distinct reinfection rates for this risk group, namely the observed reinfection rate in the Dat'AIDS cohort (Table 1) and the mean first infection rate observed between 2012 and 2015. All analyses presented here were conducted using R version 3.3.2 (R Foundation for Statistical Computing, Vienna, Austria).

## Results

### HIV-HCV patients in France

On January 1, 2016, 156,811 patients were estimated to be infected with HIV in France, of whom 24,900 (16%) were part of the undiagnosed population [19]. Using our cohort data, we estimated a total of 7216 diagnosed and under-care HIV-patients with active HCV infection and 722 HIV-HCV undiagnosed patients (Additional file 1: Table S4). High- and low-risk MSM were estimated to represent 18% and 82% of HIV-monoinfected MSM, respectively. New HCV infections in high-risk MSM increased from 137 cases in 2012 to 291 cases in 2015 (Additional file 1: Table S5), while the annual incidence rate increased in this group from 1.84% in 2012 to 3.42% in 2015 (Table 1).

### Model goodness-of-fit and model projections over the next decade in the whole HIV-HCV population

**Fig. 2:**
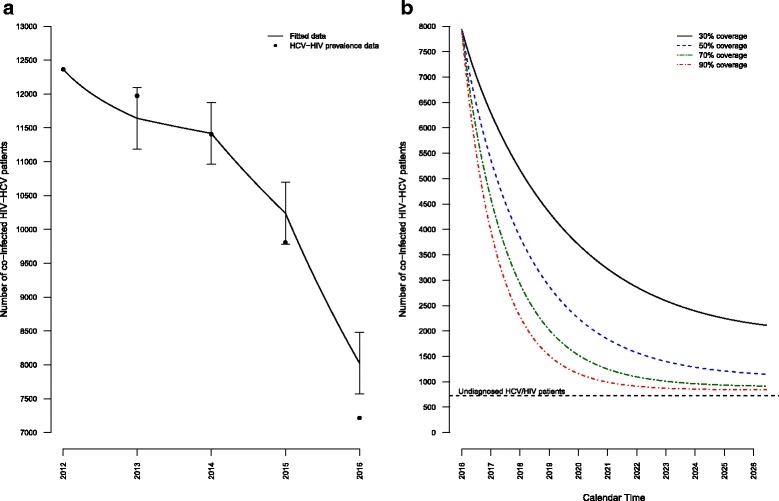
Goodness-of-fit of the compartmental model to the prevalence data between 2012 and 2016 (**a**) and projected HCV prevalence over the next 10 years in the overall HIV population (**b**). To numerically estimate the goodness-of-fit of our model, we calculated a root mean square error (RMSE) of 459 individuals between 2012 and 2016. Vertical lines indicate the RMSE for each year between 2012 and 2016. Different annual treatment coverage rates were considered for the 10-year projection (panel B): 30%, 50%, 70%, and 90%. The number of HIV-HCV coinfected patients in the undiagnosed population is represented with a dashed horizontal line. This population was not considered eligible for direct-acting antiviral treatment

Figure 2 depicts the model goodness-of-fit to the yearly prevalence data observed in the cohort between January 1, 2012, and January 1, 2016 (panel A). We observed a significant decrease of HIV-HCV coinfected individuals, in particular between 2013 and 2014 in the Dat'AIDS cohort, which follows the considering that DAA became available for all HIV-HCV coinfected patients in France in 2014 and 92% of HIV-HCV patients who started an HCV treatment received a DAA combination at that time. Mean observed SVR rate consequently increased from 70% to 89% and treatment coverage rate also increased from 6.1% in 2013 to 15.7% in 2014.

We observed an overall goodness-of-fit of the model to the yearly prevalence data. This goodness-of-fit was more precisely explored in each subgroup and we observed a similar close fit to the observed data (Additional file 3: Figure S2), except for MSM, for whom observed data were slightly below model predictions.

We then compared several treatment coverage rates and observed a significant decrease of active HCV infections among all patients over the next 10 years (Fig. 2, panel b). Assuming an annual treatment coverage of 30% (i.e., the observed 2015 treatment coverage in our cohort), the HCV prevalence in 2026 is expected to drop from 5.09% to 1.08% in the whole HIV-HCV population and from 5.48% to 0.81% in patients under care. Under this hypothesis, the number of patients with active HCV infection will decrease to 2113 patients by 2026, with 34% remaining HIV-HCV undiagnosed. Increasing treatment coverage to 50% and 70% will result in 1151 and 916 patients with active HCV infection in 2026, respectively, among whom 63% and 79% will remain undiagnosed.

### Model projections over the next decade in each risk group

**Fig. 3:**
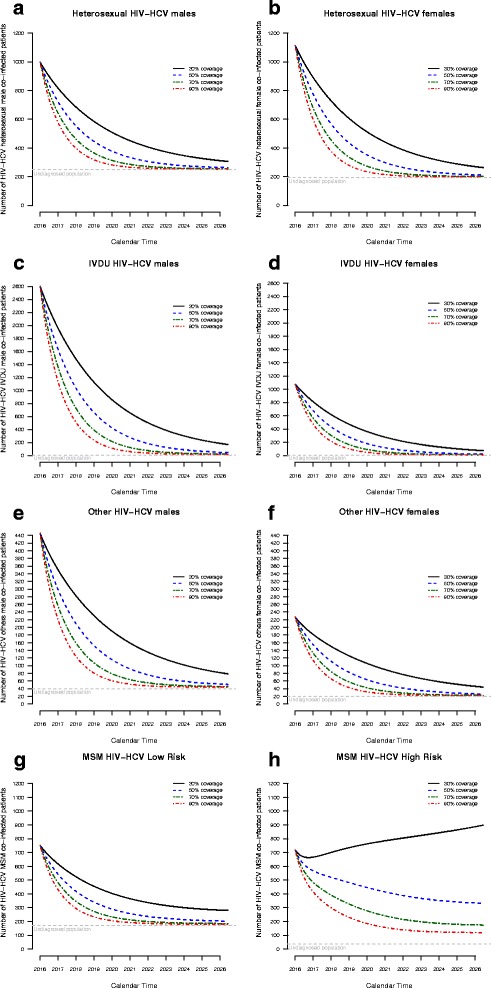
Projected prevalence (raw numbers) of HIV-HCV coinfection over the next 10 years within each risk group

We conducted similar projections for each subgroup considering several treatment coverage rates (Fig. 3, Additional file 4: Figures S3, and Additional file 5: Figure S4). We observed a similar significant decrease in predicted prevalence over the next 10 years among heterosexuals, IVDU, and patients with other risk factors for both sexes as well as for low-risk MSM. For example, prevalence of HCV infection is expected to drop from 33.1% in 2016 to 2.4% in 2026 among male IVDU under care (the largest group of patients) with a treatment coverage rate of 30%, resulting in 173 patients with active infection in this subgroup in 2026. Increasing treatment coverage rate to 50% and 70% will result in less than 100 patients in this subgroup by 2023 and 2021, respectively.

On the other hand, prevalence among under-care, high-risk MSM is predicted to slightly decrease, from 6.96% in 2016 to 6.34% in 2026, with a 30% treatment coverage rate. Meanwhile, the number of high-risk MSM with active HCV infection will increase from 719 to 839 patients in 2026. An increase in the annual treatment coverage rate to 50% or 70% would be required in this subgroup to decrease the predicted prevalence to 2.35% and 1.25% in 2026, respectively. The number of under-care, coinfected, high-risk MSM will drop below 100 cases by 2022 only if treatment coverage rate is increased up to 90%.

### Sensitivity analyses

We explored the impact of treating acute HCV infection for high-risk MSM by deriving the model structure reported in Fig. 1 (Additional file 1: Figure S1). We assumed several treatment coverage rates for acute HCV infection combined with 30% and 50% treatment coverage rates for chronic HCV infection. Assuming a 30% treatment coverage rate for chronic infection, increasing the treatment coverage rate of acute infections to 30–50% would maintain the total number of active HCV infection in high-risk MSM, while increasing acute infection treatment coverage rate to 70–90% would marginally decrease this number. Thus, with a 50% treatment coverage rate for chronic infection, the benefit of treating acute HCV infection appears marginal (Additional file 6: Figure S5).

We also explored the impact of a potential increase in the proportion of HIV monoinfected high-risk MSM over the next 10 years on HIV-HCV prevalence in this subgroup (Additional file 7: Figure S6). We considered several treatment coverage rates of chronic HCV infection and two different rates of increase in the high-risk MSM proportion, namely 5% and 10% over the next 10 years (i.e., from 18% to 23% and from 18% to 28%, respectively). We observed similar results than those reported in the main analysis, with a significant increase in the number of HIV-HCV individuals when considering an annual treatment coverage rate of 30%. On the other hand, the number of HIV-HCV high-risk MSM is expected to decrease when the annual treatment coverage rate reaches at least 50% despite an increase in the proportion of high-risk MSM in the HIV monoinfected population.

We finally investigated the impact of considering an external force of infection for IVDU to take into account the potential risk of HCV transmission between HIV-negative and HIV-positive individuals. We derived our main model and we considered several proportions of external cases among those observed in the Dat'AIDS cohort between 2012 and 2015. Estimated numbers of HIV-HCV IVDU over the next 10 years are reported in Additional file 1: Table S6 and S7 and Additional file 8: Figure S7 assuming several proportions of external cases. Under this hypothesis, the number of HIV-HCV IVDU in 2026 is expected to marginally increase compared with our main analysis, while HIV-HCV prevalence in this risk group is expected to significantly decrease over the next 10 years, even if the considered proportion of external HCV cases is high (>60% of total IVDU cases).

## Discussion

Prevalence of active HCV infection among HIV patients in France is globally projected to decrease from 5.09% to 1.08% within the next 10 years under current treatment rates. This decrease should result in approximately 2100 patients with active HCV infection in 2026, 34% of whom will remain outside of the healthcare system. In our model, a decrease of active HCV infection prevalence is expected in almost all risk groups, except for high-risk MSM in whom HCV prevalence would remain almost stable, unless a minimum of 50% treatment coverage rate is reached.

To our knowledge, this is the first study to model HCV epidemic among HIV-infected patients in a whole country and within all risk groups. Since incidence and prevalence data of HIV-HCV coinfection were heterogeneous among the different risk groups in the Dat’AIDS cohort, we considered eight distinct risk groups, including high-risk MSM, who represent 18% of all HIV-monoinfected MSM. This estimated proportion is close to the 25% estimate reported within the Swiss HIV cohort using patients’ unsafe sex reports as reference [14], while a lower estimate of 7% was reported in the UK Collaborative HIV cohort [13].

HIV-HCV coinfection was historically associated with intravenous drug use in France and this risk group remained the largest group of patients at the beginning of 2016. However, very few new HCV infections were observed in this risk group, resulting in a drastic decrease in our projections. On the other hand, HCV infection incidence rate in high-risk MSM increased in the cohort since the early 2000s and nearly doubled in this group between 2012 and 2015 [2]. This trend was also observed in the Swiss cohort [14, 22], as well as in cohorts from Netherlands [23] or Japan [24], with similar estimates, but was not observed in the UK [13]. The reinfection rate observed in our cohort was similar to a pooled reinfection rate estimate of 32/1000 person-years reported in a meta-analysis [12], while higher rates were recently reported in the UK (7.8/100 person-years) and within the European NEAT cohort (7.3/100 person-years) [9, 11]. High-risk MSM could therefore drive an HIV-HCV epidemic over the next years in France and, potentially, in other high-income countries. In order to control such a future epidemic, the targeting of high-risk behavior MSM patients will be crucial.

In our study, we considered that all HIV patients after 6 months of HCV infection were eligible for DAA treatment as recommended in France. However, we also demonstrated a marginal effect of treating high-risk MSM from the third month of infection. Martin et al. [13] explored the benefit of HCV treatment at both the chronic and acute phases of HCV infection, but defined the acute phase after 1 year of infection. Patients after 6 months of HCV infection are eligible for DAA treatment in France. It is therefore likely that a high treatment coverage for all patients after 6 months of HCV infection would have a similar effect on HCV prevalence than a combined strategy of treating HCV patients during the acute phase (before 6 months) and after 6 months for other patients, but with a lower treatment coverage for the latter. Indeed, in our projections, increasing the acute infection treatment coverage rate to over 70% in high-risk MSM slightly strengthened a decrease in HIV-HCV coinfection prevalence. Increasing HCV treatment coverage after 6 months of infection in high-risk MSM consequently appears as an effective solution to significantly decrease HCV prevalence in this population over the next 10 years.

We used estimates of the undiagnosed HIV population to analyze the impact of HCV treatment on the whole HIV-HCV epidemic in France. Our projections show that the proportion of HIV-HCV undiagnosed patients could increase to 35% in 2026 considering a 30% treatment coverage rate. This proportion could increase to 64% and 79% in 2026 with treatment coverage rates of 50% and 70%, respectively. Although our estimates of coinfection in this undiagnosed population are relatively low, undiagnosed HIV-HCV patients could fuel an HCV epidemic in the future if no specific interventions are undertaken to identify and enroll undiagnosed patients in care.

Our model does have some limitations. First, we considered that no mixing occurred between MSM, heterosexuals, IVDU, and other risk groups. While HCV transmission among heterosexual couples is rare [25], the source of HCV infection in MSM is often difficult to establish due to concomitant use of intravenous or nasal drugs, and sexual risk behavior [26]. However, drug use in MSM appears to be mostly driven by consumption during sexual intercourse, and there is no evidence that former opiate users could be a significant source of HCV infection in MSM [27–29].

Second, we modeled HCV transmission among HIV-infected patients only, without considering any other route of transmission such as from the monoinfected HCV population. In a sensitivity analysis, we considered an external force of infection for IVDU to investigate the impact of a potential risk of HCV transmission between HIV-negative and HIV-positive IVDU; the model projections under this hypothesis were similar to our main analysis projections. However, it is likely that HCV transmission between HIV-negative and HIV-positive IVDU could fuel the HCV epidemic in this risk group in other epidemiological contexts, more particularly in countries where HCV incidence and prevalence remain high in this risk group [30, 31]. Moreover, the observed reinfection rate among IVDU in the Dat'AIDS cohort was lower than the reinfection rate observed in other risk groups. Most of IVDU (90%) included in the cohort were former drug users with a median age of 55 years, which could explain why the observed reinfection rate in this risk group was relatively low. It is potentially likely that HCV reinfection rate in HIV-positive IVDU could be higher in younger, active IVDU, who may not be well represented in the Dat'AIDS cohort compared with other epidemiological context such as in the US.

On the other hand, a recent study from the Netherlands reported that similar HCV strains were circulating among HIV-HCV coinfected MSM and among MSM with high-risk behaviors engaged in a pre-exposure prophylaxis program and acutely infected by HCV [32]. Since HCV infection incidence is usually lower in HIV-negative than in HIV-positive MSM, this study suggests that HCV infection is now spreading from HIV-positive to HIV-negative MSM. Another recent study from the Netherlands showed a significant decrease in the number of acute HCV infections among HIV positive MSM since the commencement of universal access to DAA treatment for this population [33]. These results are in favor of a limited HCV transmission from HIV-negative to HIV-positive patients. In any case, a recent increase in unprotected sexual intercourse and sexually transmitted infection incidence in HIV-negative MSM as well as acute HCV infections in MSM enrolled in a pre-exposure prophylaxis program warn us to increase regular HCV screening of all high-risk MSM regardless of HIV infection [34–37].

Third, we estimated the number of undiagnosed HIV-HCV patients in France using the hypothesis of a similar HCV prevalence in this population compared with new patients entering the Dat’AIDS cohort in recent years. Therefore, we added these patients as a constant over time in the projections and no interaction between this population and HIV-diagnosed patients was considered. It is also possible that the size of this population will change in the future due to potential interventions for HIV-diagnosed patients.

Fourth, the model neglected international migration and did not consider the potential risk of HCV transmission related to MSM international networks, as previously described [38, 39], since no clear data were available to integrate this risk of HCV transmission in our model.

Fifth, we estimated the proportion of HIV-monoinfected high-risk MSM as a constant over time in our main analysis. A specific definition of this population is, to date, not globally approved. We assessed the impact of an increase in the proportion of HIV monoinfected high-risk MSM on HIV-HCV prevalence in the model projections but we did not assess the impact of effective behavioral interventions in this population because no study has thus far proved an effect of such an intervention for HCV infection on the size of the high-risk population.

Sixth, we considered the reinfection rate as a constant force of infection in each risk group (i.e., independent of HCV prevalence), as the proportion of low- and high-risk MSM could not be determined among HIV-HCV coinfected MSM, except at the beginning of the calibration process, i.e. on January 1^st^, 2012 (Additional file 1: Appendix). Finally, our projections are promising as most HIV-infected individuals in France are under care and DAA treatment access is universal, i.e., not restricted to a specific fibrosis score and/or comorbidities. These projections are likely to be different if our model was fitted to another country’s settings without universal DAA treatment access and/or with a different spectrum of engagement in HIV care.

Our study also has a number of strengths. The model is based on the largest and most exhaustive HIV-HCV database reported in the literature to date, with yearly data available for all included patients. Moreover, all risk groups were considered in the model for calibration and projections, and reliable HCV risk of transmission for the first infection in each risk group was thus estimated during the calibration by extending the observed data to the whole HIV-diagnosed population in France.

## Conclusion

Our study demonstrates that the number of active HCV infection in under-care HIV-infected patients is expected to drastically decrease within the next decade. However, an increase in new infection and reinfection incidence in high-risk MSM as well as an increase in the proportion of undiagnosed patients and occurrence of acute HCV infection in non-HIV-infected MSM could fuel an HCV epidemic in the future. Addressing all these issues is necessary to achieve HCV elimination in this population.

## Additional files

Additional file 1: **Appendix. Table S1.** Extrapolated numbers of HIV-monoinfected patients by subgroups in France in the population under care on January 1, 2016. **Table S2.** Extrapolated numbers of HIV-HCV coinfected patients with detectable HCV-RNA by subgroups in France in the population under care on January 1, 2016. **Table S3.** Extrapolated numbers of HIV-infected patients successfully treated for HCV or after spontaneous HCV clearance by subgroups in France in the population under care on January 1, 2016. **Table S4.** Estimated numbers of HIV-HCV coinfected patients by subgroup in France in the HIV undiagnosed population. **Table S5.** Number of first HCV infection observed each year in the Dat’AIDs cohort and extended to the diagnosed HIV population in France by risk group. **Table S6.** Estimated numbers of HIV-HCV coinfected IVDU considering several proportions of external cases, observed reinfection rate in the Dat'AIDs cohort, and an annual treatment coverage of 30% over the next 10 years. **Table S7.** Estimated numbers of HIV-HCV coinfected IVDU considering several proportions of external cases, reinfection rate based on mean first infection rate observed in the Dat'AIDs cohort, and an annual treatment coverage of 30% over the next 10 years. (DOCX 44 kb)

Additional file 2: Figure S1. Schematic diagram of HCV transmission compartmental model considering potential HCV treatment during acute phase. (PDF 26 kb)

Additional file 3: Figure S2. Goodness of fit of the compartmental model to prevalence data between 2012 and 2016 in each risk group (heterosexuals, IVDU, MSM and others). (PDF 34 kb)

Additional file 4: Figure S3. Projected prevalence of HIV-HCV coinfection over the next 10 years within each risk group assuming an annual treatment coverage of 30%. (PDF 1673 kb)

Additional file 5: Figure S4. Projected prevalence (rate) of HIV-HCV coinfections over the next 10 years within each risk groups. (PDF 528 kb)

Additional file 6: Figure S5. Projected prevalence of HIV-HCV coinfections over the next 10 years considering potential HCV treatment during acute phase among high risk MSM. (PDF 187 kb)

Additional file 7: Figure S6. Projected prevalence of HIV-HCV coinfections over the next 10 years considering a linear increase of the proportion of high risk HIV monoinfected. (PDF 148 kb)

Additional file 8: Figure S7. Projected prevalence of HIV-HCV coinfections over the next 10 years in IVDU considering a potential risk of HCV transmission between HIV-negative and HIV-positive individuals. (PDF 153 kb)
