# Supplementary material for: Modeling HIV-HCV coinfection epidemiology in the direct-acting antiviral era: the road to elimination
Source: BMC Med. 2017 Dec 18;15:217. doi: 10.1186/s12916-017-0979-1 (PMC5733872; doi:10.1186/s12916-017-0979-1)
Supplement: Supplementary file 1 — Appendix. Table S1. Extrapolated numbers of HIV-monoinfected patients by subgroups in France in the population under care on January 1, 2016. Table S2. Extrapolated numbers of HIV-HCV coinfected patients with detectable HCV-RNA by subgroups in France in the population under care on January 1, 2016. Table S3. Extrapolated numbers of HIV-infected patients successfully treated for HCV or after spontaneous HCV clearance by subgroups in France in the population under care on January 1, 2016. Table S4. Estimated numbers of HIV-HCV coinfected patients by subgroup in France in the HIV undiagnosed population. Table S5. Number of first HCV infection observed each year in the Dat’AIDs cohort and extended to the diagnosed HIV population in France by risk group. Table S6. Estimated numbers of HIV-HCV coinfected IVDU considering several proportions of external cases, observed reinfection rate in the Dat'AIDs cohort, and an annual treatment coverage of 30% over the next 10 years. Table S7. Estimated numbers of HIV-HCV coinfected IVDU considering several proportions of external cases, reinfection rate based on mean first infection rate observed in the Dat'AIDs cohort, and an annual treatment coverage of 30% over the next 10 years. (DOCX 44 kb) [file 12916_2017_979_MOESM1_ESM.docx]

**Additional file 1**

**Modeling HIV-HCV coinfection epidemiology in the DAA era: 
the road to elimination**

Victor Virlogeux, Fabien Zoulim, Pascal Pugliese, Isabelle Poizot-Martin, Marc-Antoine Valantin, Lise Cuzin, Jacques Reynes, Eric Billaud, Thomas Huleux, Firouze Bani-Sadr, David Rey, Anne Frésard, Christine Jacomet, Claudine Duvivier, Antoine Cheret, Laurent Hustache-Mathieu, Bruno Hoen, André Cabié, Laurent Cotte and the Dat’AIDS study Group

**Table of contents**

Hepatitis C transmission model…………………………………………………………………2

Calibration process and estimation of parameters……………………………………...3

Sensitivity Analyses…………………………………………………………………………….........5

Supplementary Table 1……………………………………………………………………………..8

Supplementary Table 2……………………………………………………………………………..9

Supplementary Table 3………………………………………………………..…………………..10

Supplementary Table 4………………………………………………………..…………………..11

Supplementary Table 5…………………………………………………………..………………..12

Supplementary Table 6…………………………………………………………..………………..13

Supplementary Table 7…………………………………………………………..………………..14

Supplementary Figure 1……………………………………………………………….……..…...15

Supplementary Figure 2………………………………………………………..………………....16

Supplementary Figure 3………………………………………………………..………………....17

Supplementary Figure 4………………………………………………………..………………....18

Supplementary Figure 5………………………………………………………..………………....19

Supplementary Figure 6………………………………………………………..………………....20

Supplementary Figure 7………………………………………………………..………………....21

**Hepatitis C transmission model**

To model HCV transmission among HIV patients we designed a compartmental model defined by the following system of ordinary differential equations (see Figure 1 for the schematic diagram of HCV transmission compartmental model):

$$\frac{dX_{j}}{dt}=\theta_{j}-\beta_{j}X_{j}(t)\overline{I}(t)-\mu X_{j}(t)$$

$$\frac{dA_{j}}{dt}=\beta_{j}X_{j}(t)\overline{I}(t)-\gamma\psi A_{j}(t)+\delta_{j}S_{j}(t)-\left( 1-\gamma\right)\psi A_{j}(t)-\mu A_{j}(t)$$

$$\frac{{dC}_{j}}{dt}=\left( 1-\gamma\right)\psi A_{j}(t)-\tau C_{j}(t)+\left( 1-\alpha\right)\omega T_{j}(t)-\mu C_{j}(t)$$

$$\frac{dT_{j}}{dt}=-\alpha\omega T_{j}(t)-\mu T_{j}(t)-\left( 1-\alpha\right)\omega T_{j}(t)+\tau C_{j}(t)$$

$$\frac{dS_{j}}{dt}=\alpha\omega T_{j}(t)-\delta_{j}S_{j}(t)+\gamma\psi A_{j}(t)-\mu S_{j}(t)$$

where :

$\overline{I}\left( t \right)=A_{Mlr}+A_{Mhr}+C_{Mlr}+C_{Mhr}$ with *Mlr* for MSM low-risk and *Mhr* for MSM high-risk when *j=Mlr* or *j=Mhr*

$\overline{I}\left( t \right)=A_{Hetero\_males}+A_{Hetero\_females}+C_{Hetero\_males}+C_{Hetero\_females}$ when *j=Hetero_males* or *j=Hetero_females*

$\overline{I}\left( t \right)=A_{IVDU\_males}+A_{IVDU\_females}+C_{IVDU\_males}+C_{IVDU\_females}$ when *j=IVDU_males* or *j=IVDU_females*

$\overline{I}\left( t \right)=A_{Ohers\_males}+A_{Others\_females}+C_{Others\_males}+C_{Ohers\_females}$ when *j=Others_males* or *j=Others_females*

**Calibration and estimation of parameters**

We calibrated our model using yearly observed incidence and prevalence data (raw numbers) in the Dat’AIDS cohort from January 1^st^, 2012 to January 1^st^, 2016. Infection rate β_j_ (first infection) was estimated in each subgroup *j*. To estimate these parameters, we used a Poisson-based likelihood defined by:

$$L\left( \beta_{j},{Data}_{incidence},{Data}_{prevalence} \right)= \prod_{i=2012}^{i=2016} P\left( X_{i,j},E(X_{i,j}) \right)\times P\left( Y_{i,j},E(Y_{i,j}) \right)$$

where:

X_i,j_=number of new first HCV infection in subgroup *j* during the year *i* (Data_incidence_)

Y_i,j_= number of co-infected patients (A_j_+C_j_) in the subgroup *j* on the 1^st^ January of year *i+1* (Data_prevalence_)

$E\left( X_{i,j} \right)=\sum_{t=1}^{t=365} \beta\overline{I}(t)X_{j}(t)$ for each year *i* in each subgroup *j*

$E\left( Y_{i,j} \right)=\overline{I}(t=01/01 of year i+1)$ in each subgroup *j*

P is the probability density function of a Poisson distribution, with a mean E(X_i_):

$P\left( X_{i},E(X_{i}) \right)=\frac{{E(X_{i})}^{X_{i}}\times exp\left( -E(X_{i}) \right)}{X_{i}!}$

**Bayesian framework**

In order to estimate these different parameters, we used a Bayesian framework with a Monte Carlo Markov Chain Method (MCMC). In this framework, if $\theta$ represents a vector of parameters and $y$ the data, Bayes theorem gives us the following relationship:

$$\boldsymbol{p}\left( \boldsymbol{\theta} | \boldsymbol{y} \right)\boldsymbol{=}\frac{\boldsymbol{p}\left( \boldsymbol{y} | \boldsymbol{\theta} \right)\boldsymbol{p}\left( \boldsymbol{\theta} \right)}{\boldsymbol{p}\left( \boldsymbol{y} \right)}$$

where $p\left( \theta\right)$ is the prior probability of the parameters $\theta$, $p\left( y | \theta\right)$ is the likelihood function and $p\left( \theta| y \right)$ is the posterior probability of $\theta$ given the data $y$.

MCMC process was initiated by giving random values to the parameters $\theta$ and by choosing non-informative prior (flat prior) for $\theta$, with a normal prior for all parameters, *N*(0,10000). A Metropolis Hastings algorithm was used to update the parameter values in each iteration. In each iteration, all the $k$ parameters are randomly generated using the normal distribution with the mean $\theta_{k}^{j-1}$ (previous value of the k^th^ parameter) and standard error $\sigma_{k}$ , $N(\theta_{k}^{j-1},\sigma_{k}$ ) for each parameter. The updated likelihood is compared with the previous one using the following accept-reject method:

$$\boldsymbol{q=}\frac{\boldsymbol{p}\left( \boldsymbol{y} | \theta^{j} \right)\boldsymbol{p}\left( \theta^{j} \right)}{\boldsymbol{p}\left( \boldsymbol{y} | \theta^{j-1} \right)\boldsymbol{p}\left( \theta^{j-1} \right)}$$

If $q\geq1$, the proposed new values of parameters $\theta^{j}$ are accepted

If $q<1$, then $\theta^{j}$ are accepted with probability $q$.

The above algorithm was repeated 10,000 times after a burn-in period of 5,000 repetitions, with an acceptance rate included in $\left[ 0.45, 0.55 \right]$ for each parameter*.* To check the quality of the different runs, we used visual inspection of sampling, the Gelman-Rubin and the Geweke diagnostic tests using the R packages "MCMCpack" and "coda".

To numerically estimate the goodness-of-fit of our model to the prevalence data, we calculate the root mean square error (RMSE) using the R package “hydroGOF”.

**Estimation of the proportion of monoinfected high-risk MSM**

To estimate the proportion of high-risk MSM, we assumed that the reinfection rate observed in MSM was representative of the first infection rate in this subgroup. On the other hand, we assumed that the first infection rate of low-risk MSM was equal to the first infection rate observed in other risk groups. We calculated the proportion of high-risk MSM *p* each year between 2012 and 2015 using the following equation and we used the mean proportion in our model (p=0.18):

$$p=\frac{{incidence}_{All MSM}-{incidence}_{Low-risk MSM}}{{incidence}_{High-risk MSM}-{incidence}_{Low-risk MSM}}$$

**Estimation of the proportion of coinfected high-risk MSM on January 1^st^, 2012**

To estimate the proportion of coinfected high-risk MSM on January 1^st^, 2012, we used yearly first infection and reinfection incidence rate data, SVR rates and treatment coverage rates from the DAT’AIDS cohort from January 1^st^, 2003 to January 1^st^, 2012. Using the same compartmental model for MSM only and a similar Poisson-based likelihood method using MSM coinfection prevalence data, we estimated a linear increase of the proportion of high-risk MSM entering the monoinfected status from 3% in 2003 to 18% in 2012 and we estimated a proportion of high-risk coinfected MSM of 20% on January1^st^, 2012.

**Sensitivity Analyses: Impact of considering an external force of HCV infection among IVDU**

We investigated the impact of considering a potential external source of HCV transmission for IVDU, i.e. from HIV-negative to HIV-positive IVDU, on our model projections over the next ten years. We derived our main model by adding a constant external force of infection (i.e. independent of HCV prevalence among HIV-positive IVDU) among IVDU.

To estimate this external force of infection, we considered several rates *p* (20%, 40%, 60%, 80% and 100%) of potentially observed external HCV cases among IVDU in the Dat'AIDs cohort. For each rate *p* of external HCV cases among IVDU*,* we re-estimated: (i) the number of new HCV cases per year resulting from HCV transmission within the HIV-positive IVDU (i.e. $(1-p)\times N_{total number of yearly observed HCV cases among IVDU}$) and (ii) the number of new HCV cases per year resulting from HCV transmission between HIV-negative and HIV-positive IVDU (i.e. $p\times N_{total number of yearly observed HCV cases among IVDU}$).

We therefore could estimate the annual external HCV infection rate for each calendar year between 2012 and 2015 using the estimated raw number of "external HCV cases" as described above. In our model, we used the mean annual external HCV infection rate between 2012 and 2015 and the estimates are reported in Supplementary Tables 6 and 7 for each considered rate *p* of "external HCV cases".

Regarding the internal force of infection among IVDU (i.e. within the HIV-positive IVDU population) we calibrated again our model using the adjusted yearly observed incidence data (i.e. taking into account only the "internal HCV cases") and the prevalence data observed in the Dat'AIDs cohort from January 1st, 2012 to January 1st, 2016 using a similar Poisson-based likelihood and Bayesian framework as described above.

We also considered two distinct reinfection rates in this sensitivity analysis: the observed reinfection rate in the Dat'AIDs cohort (Table 1) and the mean first infection rate observed between 2012 and 2015 (i.e. 0.96% per year).

In this sensitivity analysis, we used the following system of ordinary differential equations for IVDUs (please see Table 2 for more details regarding the parameters):

$$\frac{dX}{dt}=\theta-(\beta I(t)+\varepsilon)X(t)-\mu X(t)$$

$$\frac{dA}{dt}=(\beta I(t)+\varepsilon)X(t)-\gamma\psi A(t)+\delta S(t)-\left( 1-\gamma\right)\psi A(t)-\mu A(t)$$

$$\frac{dC}{dt}=\left( 1-\gamma\right)\psi A(t)-\tau C(t)+\left( 1-\alpha\right)\omega T(t)-\mu C(t)$$

$$\frac{dT}{dt}=-\alpha\omega T(t)-\left( 1-\alpha\right)\omega T(t)+\tau C(t)-\mu T(t)$$

$$\frac{dS}{dt}=\alpha\omega T(t)-\delta S(t)+\gamma\psi A(t)-\mu S(t)$$

where :

- ε is the external force of HCV infection among IVDUs, estimated for each rate *p* of "external HCV cases" (see Supplementary Tables 6 and 7).

A similar calibration process than the one described above was used to estimate the internal force of infection β using prevalence and adjusted incidence data from the Dat'AIDs cohort.

**Table S1. Extrapolated numbers of HIV-monoinfected patients by subgroups in France in the population under care on January 1^st^, 2016.**

| **Sex** | **MSM** | **Heterosexual** | **IVDU** | **Others** | **Total** |
| --- | --- | --- | --- | --- | --- |
| **Female** | 0 | 29440 | 337 | 4045 | 33822 |
| **Male** | LR*: 40173  HR*: 8819 | 22248 | 786 | 6292 | 78318 |
| **Total** | 48992 | 51688 | 1123 | 10337 | 112140 |

*LR : low-risk ; HR : high-risk*

** a proportion of 18% of high-risk MSM was estimated using the assumption that the mean reinfection incidence observed between 2012 and 2015 was representative of the first infection incidence among high-risk MSM, and that low-risk MSM had a similar first HCV infection incidence than the mean incidence observed in other risk groups.*

| **Gender** | **MSM** | **Heterosexual** | **IVDU** | **Others** | **Total** |
| --- | --- | --- | --- | --- | --- |
| **Female** | 0 | 921 | 1072 | 207 | 2200 |
| **Male** | LR*: 580  HR*: 681 | 751 | 2598 | 406 | 5016 |
| **Total** | 1261 | 1672 | 3670 | 613 | 7216 |

**Table S2. Extrapolated numbers of HIV-HCV coinfected patients with detectable HCV-RNA by subgroups in France in the population under care on January 1^st^, 2016.**

*LR : low-risk ; HR : high-risk*

** A proportion of 54% of coinfected HR MSM was estimated during the calibration process*

**Table S3. Extrapolated numbers of HIV-infected patients successfully treated for HCV or after spontaneous HCV clearance by subgroups in France in the population under care on January 1^st^, 2016.**

| **Sex** | **MSM** | **Heterosexual** | **IVDU** | **Others** | **Total** |
| --- | --- | --- | --- | --- | --- |
| **Female** | 0 | 1381 | 1589 | 302 | 3272 |
| **Male** | LR*: 1536  HR*: 791 | 1154 | 4460 | 1050 | 8991 |
| **Total** | 2327 | 2535 | 6049 | 1352 | 12263 |

*LR: low-risk; HR: high-risk*

** A proportion of 34% of successfully treated or with spontaneous HCV clearance in HR MSM was estimated during the calibration process.*

**Table S4. Estimated numbers* of HIV-HCV coinfected patients by subgroup in France in the HIV undiagnosed population.**

| **Sex** | **MSM** | **Heterosexual** | **IVDU** | **Others** | **Total** |
| --- | --- | --- | --- | --- | --- |
| **Female** | 0 | 193 | 4 | 20 | 217 |
| **Male** | LR^†^: 172  HR^†^: 38 | 248 | 7 | 40 | 505 |
| **Total** | 210 | 441 | 11 | 60 | 722 |

*LR: low-risk; HR: high-risk*

******To estimate the number of coinfected patients in the undiagnosed HIV population, we assumed that HCV prevalence in this population was similar to the observed HCV prevalence among patients that are yearly included in the DAT’AIDs cohort with a mean of 2*.*9% between 2012-2015. We therefore estimated the number of co-infected patients in each subgroup using the estimates of the overall HIV undiagnosed population divided into subgroups reported by Supervie et al. AIDS 2014.*

^†^ A proportion of 18% of HR MSM was considered in this population as observed in the monoinfected HIV population

**Table S5. Number of first HCV infection observed each year in the DAT’AIDs cohort and extended to the diagnosed HIV population in France by risk group.**

|  | **MSM (low-risk/high-risk)** | **Heterosexual** | **IVDU** | **Others** |
| --- | --- | --- | --- | --- |
| **2012** |  |  |  |  |
| Female | 0 | 4 | 0 | 3 |
| Male | 7/137* | 3 | 0 | 5 |
| **2013** |  |  |  |  |
| Female | 0 | 8 | 0 | 10 |
| Male | 21/173* | 6 | 0 | 15 |
| **2014** |  |  |  |  |
| Female | 0 | 19 | 6 | 8 |
| Male | 44/195* | 15 | 15 | 21 |
| **2015** |  |  |  |  |
| Female | 0 | 15 | 5 | 7 |
| Male | 35/291* | 11 | 14 | 10 |

**For high-risk MSM, we estimated the yearly incidence rate by combining the overall incidence rate among MSM, the incidence rate among low-risk MSM and the mean proportion of 18%. From 2012 to 2015, we estimated for high-risk MSM annual incidence rates of 1*.*84% (2012), 2.23% (2013), 2*.*40% (2014) and 3*.*42% (2015). For low-risk MSM, the observed mean incidence rates in all other subgroups were 0*.*02% (2012), 0*.*06% (2013), 0*.*12% (2014) and 0*.*09% (2015). To fit our model, we used the incidence data reported as raw numbers in this table.*

**Table S6. Estimated numbers of HIV-HCV coinfected IVDU considering several proportions of external cases, observed reinfection rate in the Dat'AIDs cohort and an annual treatment coverage of 30% over the next ten years.**

| **Proportion of external cases** | **0%** | **20%** | **40%** | **60%** | **80%** | **100%** |
| --- | --- | --- | --- | --- | --- | --- |
| **Annual external infection rate* (%)** | **0** | **0.19** | **0.39** | **0.60** | **0.79** | **0.96** |
| **2016** | 3670 | 3670 | 3670 | 3670 | 3670 | 3670 |
| **2021** | 870 | 872 | 873 | 875 | 876 | 876 |
| **2026** | 232 | 238 | 244 | 249 | 253 | 257 |

**The external infection rate was averaged using incidence data of the Dat'AIDs cohort between 2012 and 2015 and adjusted on the proportion of potentially observed external cases*

**Table S7. Estimated numbers of HIV-HCV coinfected IVDU considering several proportions of external cases, reinfection rate based on mean first infection rate observed in the Dat'AIDs cohort and an annual treatment coverage of 30% over the next ten years.**

| **Proportion of external cases** | **0%** | **20%** | **40%** | **60%** | **80%** | **100%** |
| --- | --- | --- | --- | --- | --- | --- |
| **Annual external infection rate* (%)** | **0** | **0.19** | **0.39** | **0.60** | **0.79** | **0.96** |
| **2016** | 3670 | 3670 | 3670 | 3670 | 3670 | 3670 |
| **2021** | 995 | 997 | 999 | 1000 | 1001 | 1001 |
| **2026** | 408 | 412 | 418 | 423 | 428 | 431 |

**The external infection rate was averaged using incidence data of the Dat'AIDs cohort between 2012 and 2015 and adjusted on the different proportions of potentially observed external cases.*

*Reinfection rate was assumed here to be similar to the mean first infection rate observed in IVDU in the Dat'AIDs cohort between 2012 and 2015, i.e. 0.96% per year.*

**Figure S1. Schematic diagram of HCV transmission compartmental model considering potential HCV treatment during acute phase.** New individuals enter the susceptible mono-infected categories (**X**) at a rate θ ^18^.

Susceptible individuals may be acutely infected (**A**) at an infection rate *β_j_* (estimated during the calibration process for each subgroup) and the incubation period stage (**I**) will last an average 1/σ. Individuals acutely infected may progress to HCV chronic infection (**C**) at a rate (1-*γ* ) or clear spontaneously their infection at a rate *γ* (**S**) or start HCV treatment at an annual rate *τ2*. Acute infection lasts an average *1/ψ.* For chronically infected individuals, they start an HCV treatment at an annual rate *τ1.* Treatment lasts an average *1/ω* and results in SVR12 for a proportion *α* of all treated patients. Successfully treated patients or patients with spontaneous clearance may be reinfected at a re-infection rate *δ_j_*.

**Figure S2. Goodness of fit of the compartmental model to prevalence data between 2012 and 2016 in each risk group (heterosexuals, IVDU, MSM and others).** To estimate the goodness-of-fit of our model for the 4 different risk groups between 2012 and 2016, we calculated a root mean square error (RMSE) of 57 individuals for heterosexuals, of 148 individuals for IVDU, of 254 individuals for MSM and of 65 individuals for others. Vertical lines indicate the RMSE for each risk group.

**Figure S3. Projected prevalence of HIV-HCV co-infection over the next 10 years within each risk group assuming an annual treatment coverage of 30%.** Grey bands indicate 95% credibility intervals

**Figure S4.** **Projected prevalence (rate) of HIV-HCV co-infections over the next 10 years within each risk groups.**

**Figure S5. Projected prevalence of HIV-HCV co-infections over the next 10 years considering potential HCV treatment during acute phase among high risk MSM.** Panel (A) shows HIV-HCV prevalence projections considering an annual treatment coverage of chronic HCV patients of 30% and different annual treatment coverage for acute HCV patients (30%, 50%, 70% and 90%). Panel (B) shows HIV-HCV prevalence projections considering an annual treatment coverage of chronic HCV patients of 50% and different annual treatment coverage for acute HCV patients (30%, 50%, 70% and 90%).

**Figure S6.** **Projected prevalence of HIV-HCV co-infections over the next 10 years considering a linear increase of the proportion of high risk HIV monoinfected.** Panel (A) shows HIV-HCV prevalence projections considering different annual treatment coverage of chronic HCV patients of 30%, 50%, 70% and 90% and considering a 5% linear increase of the proportion of high risk HIV monoinfected over the next ten years. Panel (B) shows HIV-HCV prevalence projections considering different annual treatment coverage of chronic HCV patients of 30%, 50%, 70% and 90% and considering a 10% linear increase of the proportion of high risk HIV monoinfected over the next ten years.

**Figure S7.** **Projected prevalence of HIV-HCV co-infections over the next 10 years in IVDU considering a potential risk of HCV transmission between HIV-negative and HIV-positive individuals.**

Panel (A) shows HIV-HCV prevalence projections considering: different proportion of external cases: 0%, 20%, 60% and 100% and the mean reinfection rate observed in the Dat'AIDs cohort between 2012 and 2015. Panel (B) shows HIV-HCV prevalence projections considering different proportion of external cases: 0%, 20%, 60% and 100% and the assumption that the reinfection rate is similar to the mean first infection rate observed in the Dat'AIDs cohort between 2012 and 2015.
