# Supplementary figures and images for: Modeling HIV-HCV coinfection epidemiology in the direct-acting antiviral era: the road to elimination
Source: BMC Med. 2017 Dec 18;15:217. doi: 10.1186/s12916-017-0979-1 (PMC5733872; doi:10.1186/s12916-017-0979-1)

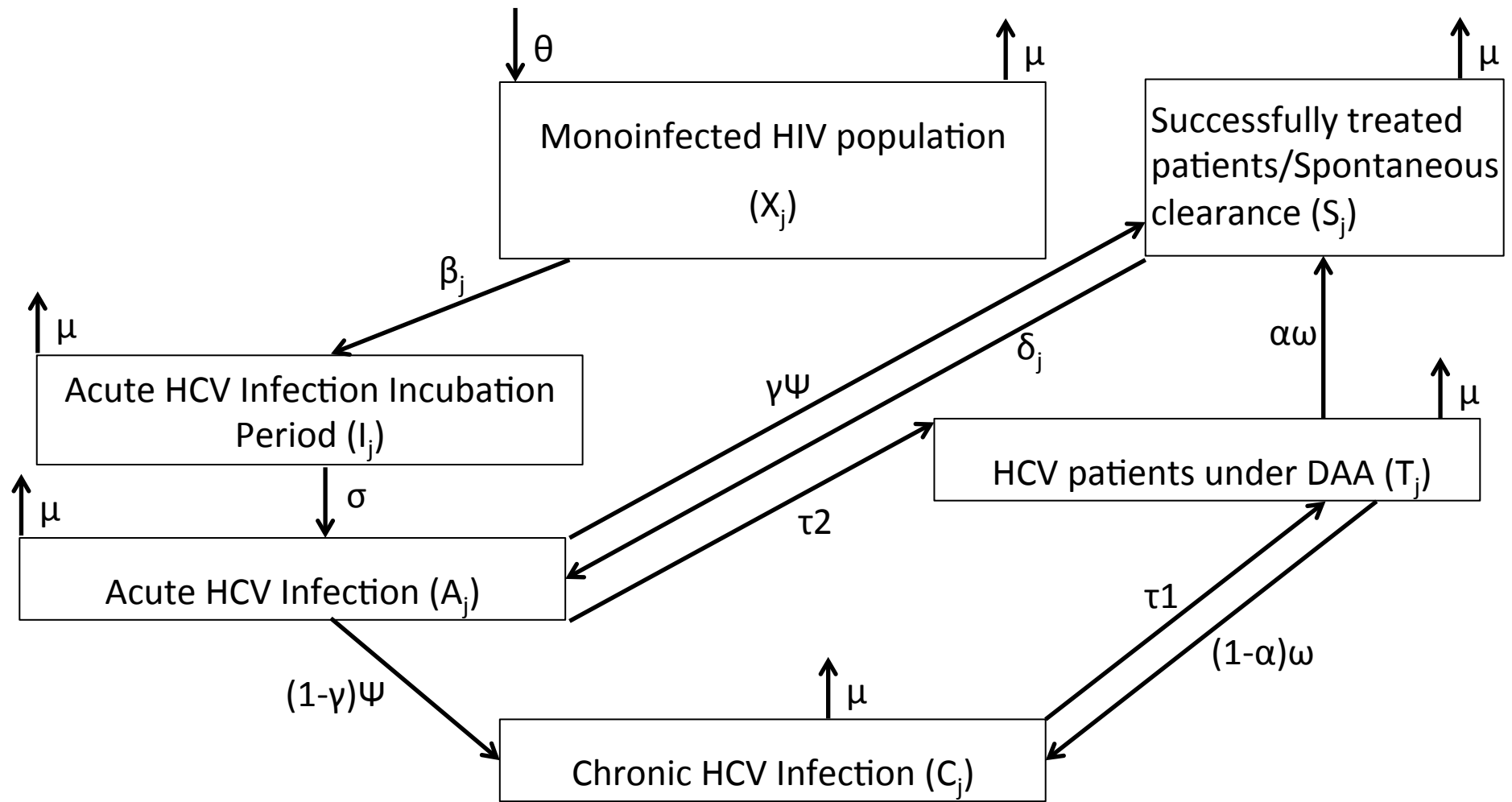

Supplement: Supplementary file 2 — Schematic diagram of HCV transmission compartmental model considering potential HCV treatment during acute phase. (PDF 26 kb) [file 12916_2017_979_MOESM2_ESM.pdf]

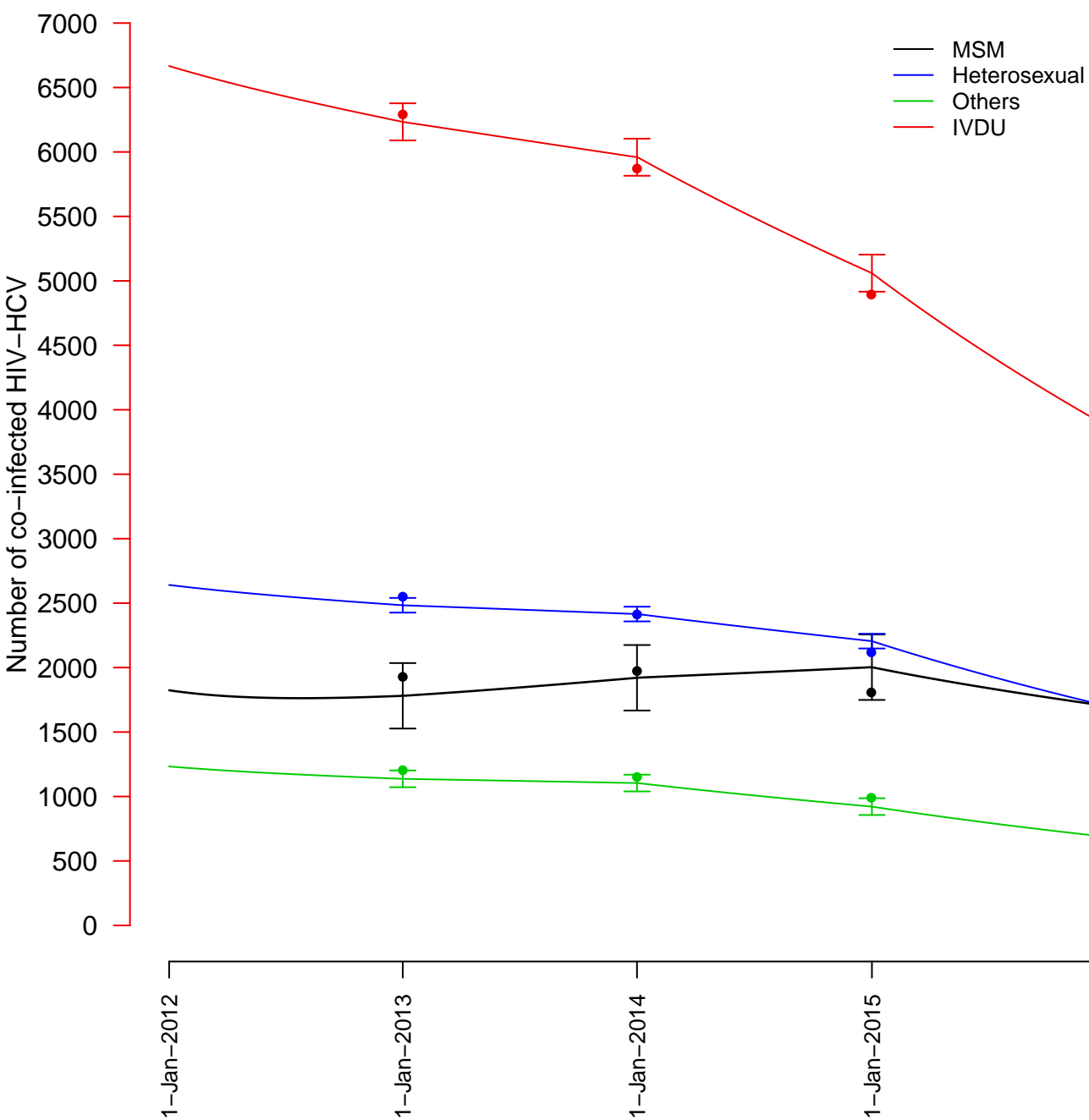

Supplement: Supplementary file 3 — Goodness of fit of the compartmental model to prevalence data between 2012 and 2016 in each risk group (heterosexuals, IVDU, MSM and others). (PDF 34 kb) [file 12916_2017_979_MOESM3_ESM.pdf]

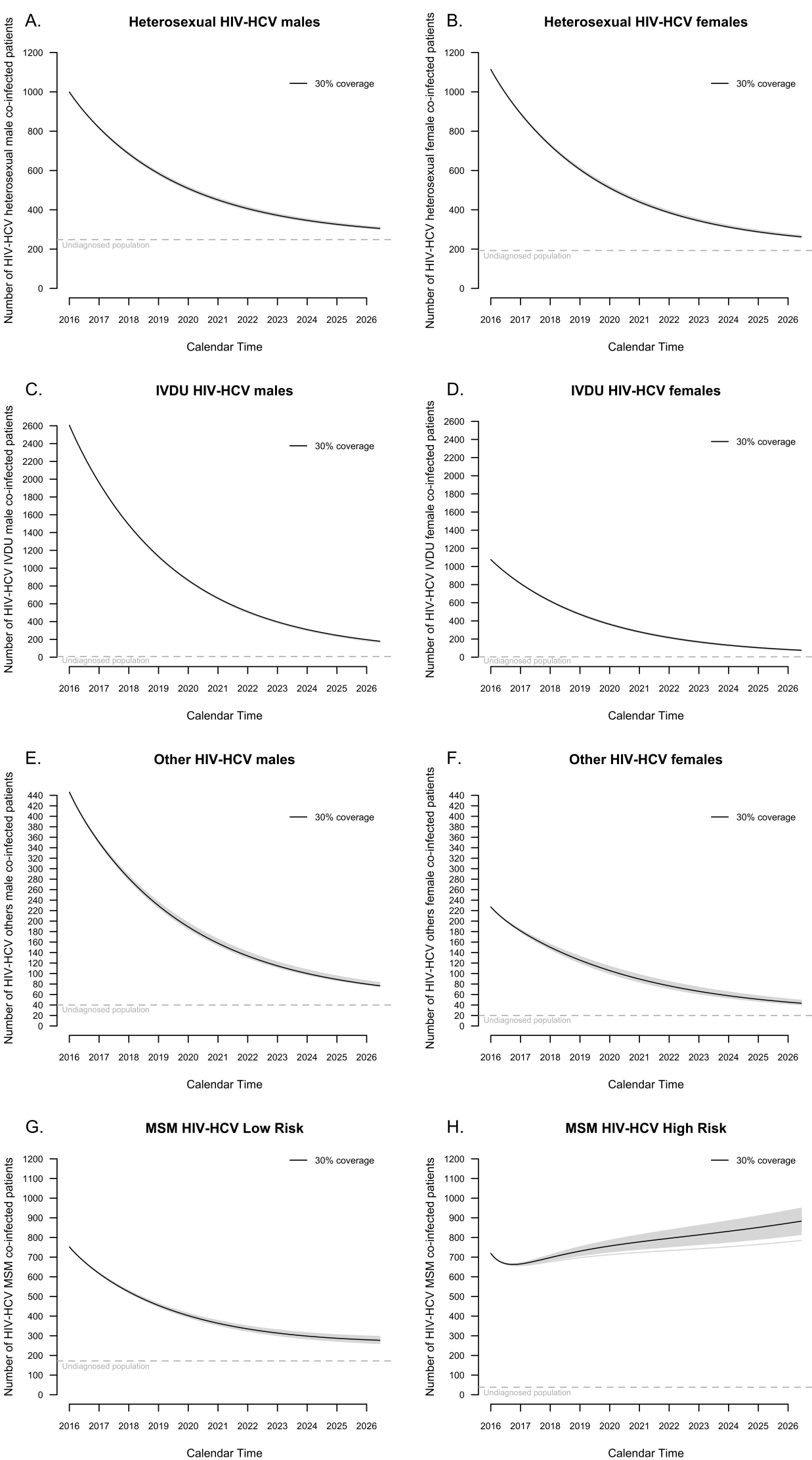

Supplement: Supplementary file 4 — Projected prevalence of HIV-HCV coinfection over the next 10 years within each risk group assuming an annual treatment coverage of 30%. (PDF 1673 kb) [file 12916_2017_979_MOESM4_ESM.pdf]
