# Supplementary material for: Modeling HIV-HCV coinfection epidemiology in the direct-acting antiviral era: the road to elimination
Source: BMC Med. 2017 Dec 18;15:217. doi: 10.1186/s12916-017-0979-1 (PMC5733872; doi:10.1186/s12916-017-0979-1)
Supplement: Supplementary file 5 — Projected prevalence (rate) of HIV-HCV coinfections over the next 10 years within each risk groups. (PDF 528 kb) [file 12916_2017_979_MOESM5_ESM.pdf]

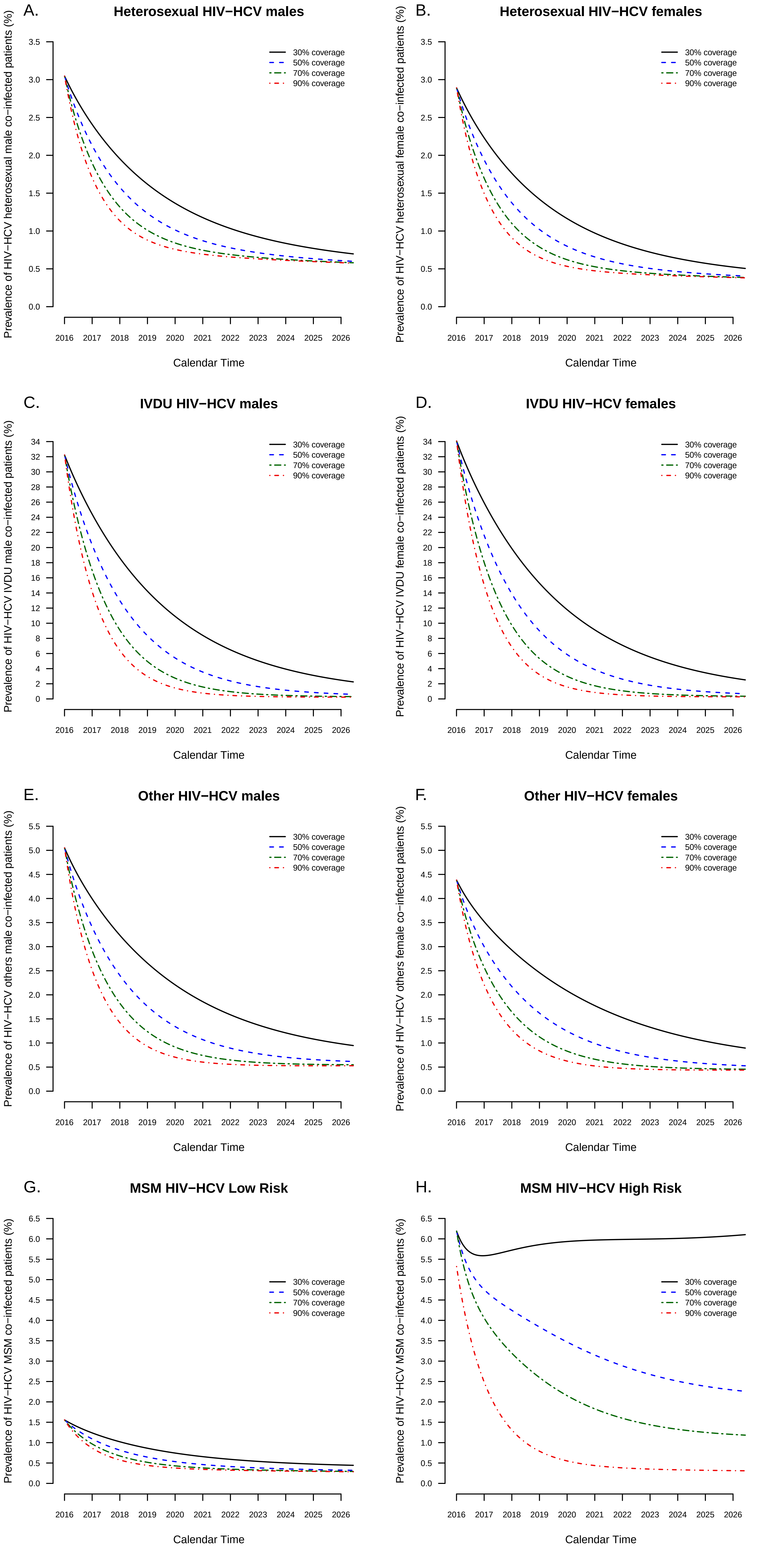
A.
Heterosexual HIV−HCV males
Prevalence of HIV−HCV heterosexual male co−infected patients (%)
0.0
0.5
1.0
1.5
2.0
2.5
3.0
3.5
2016
2017
2018
2019
2020
2021
2022
2023
2024
2025
2026
Calendar Time
30% coverage
50% coverage
70% coverage
90% coverage
B.
Heterosexual HIV−HCV females
Prevalence of HIV−HCV heterosexual female co−infected patients (%)
C.
IVDU HIV−HCV males
Prevalence of HIV−HCV IVDU male co−infected patients (%)
0
2
4
6
8
10
12
14
16
18
20
22
24
26
28
30
32
34
D.
IVDU HIV−HCV females
Prevalence of HIV−HCV IVDU female co−infected patients (%)
E.
Other HIV−HCV males
Prevalence of HIV−HCV others male co−infected patients (%)
4.0
4.5
5.0
5.5
F.
Other HIV−HCV females
Prevalence of HIV−HCV others female co−infected patients (%)
G.
MSM HIV−HCV Low Risk
Prevalence of HIV−HCV MSM co−infected patients (%)
6.0
6.5
H.
MSM HIV−HCV High Risk
