# Supplementary material for: Modeling HIV-HCV coinfection epidemiology in the direct-acting antiviral era: the road to elimination
Source: BMC Med. 2017 Dec 18;15:217. doi: 10.1186/s12916-017-0979-1 (PMC5733872; doi:10.1186/s12916-017-0979-1)
Supplement: Supplementary file 6 — Projected prevalence of HIV-HCV coinfections over the next 10 years considering potential HCV treatment during acute phase among high risk MSM. (PDF 187 kb) [file 12916_2017_979_MOESM6_ESM.pdf]

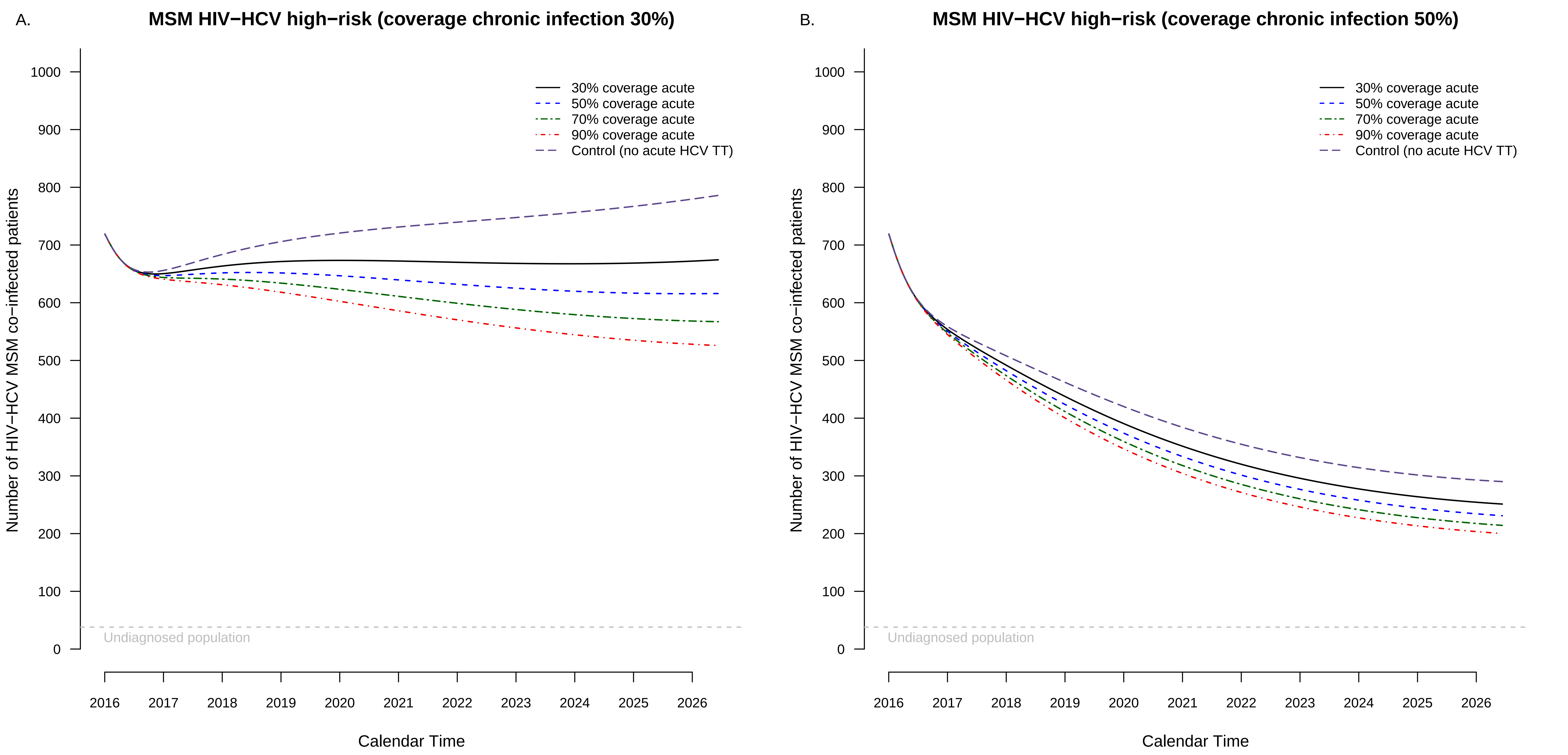
A.
MSM HIV−HCV high−risk (coverage chronic infection 30%)
B.
MSM HIV−HCV high−risk (coverage chronic infection 50%)
30% coverage acute
50% coverage acute
70% coverage acute
90% coverage acute
Control (no acute HCV TT)
Number of HIV−HCV MSM co−infected patients
0
100
200
300
400
500
600
700
800
900
1000
Undiagnosed population
2016
2017
2018
2019
2020
2021
2022
2023
2024
2025
2026
Calendar Time
