# Supplementary material for: Modeling HIV-HCV coinfection epidemiology in the direct-acting antiviral era: the road to elimination
Source: BMC Med. 2017 Dec 18;15:217. doi: 10.1186/s12916-017-0979-1 (PMC5733872; doi:10.1186/s12916-017-0979-1)
Supplement: Supplementary file 7 — Projected prevalence of HIV-HCV coinfections over the next 10 years considering a linear increase of the proportion of high risk HIV monoinfected. (PDF 148 kb) [file 12916_2017_979_MOESM7_ESM.pdf]

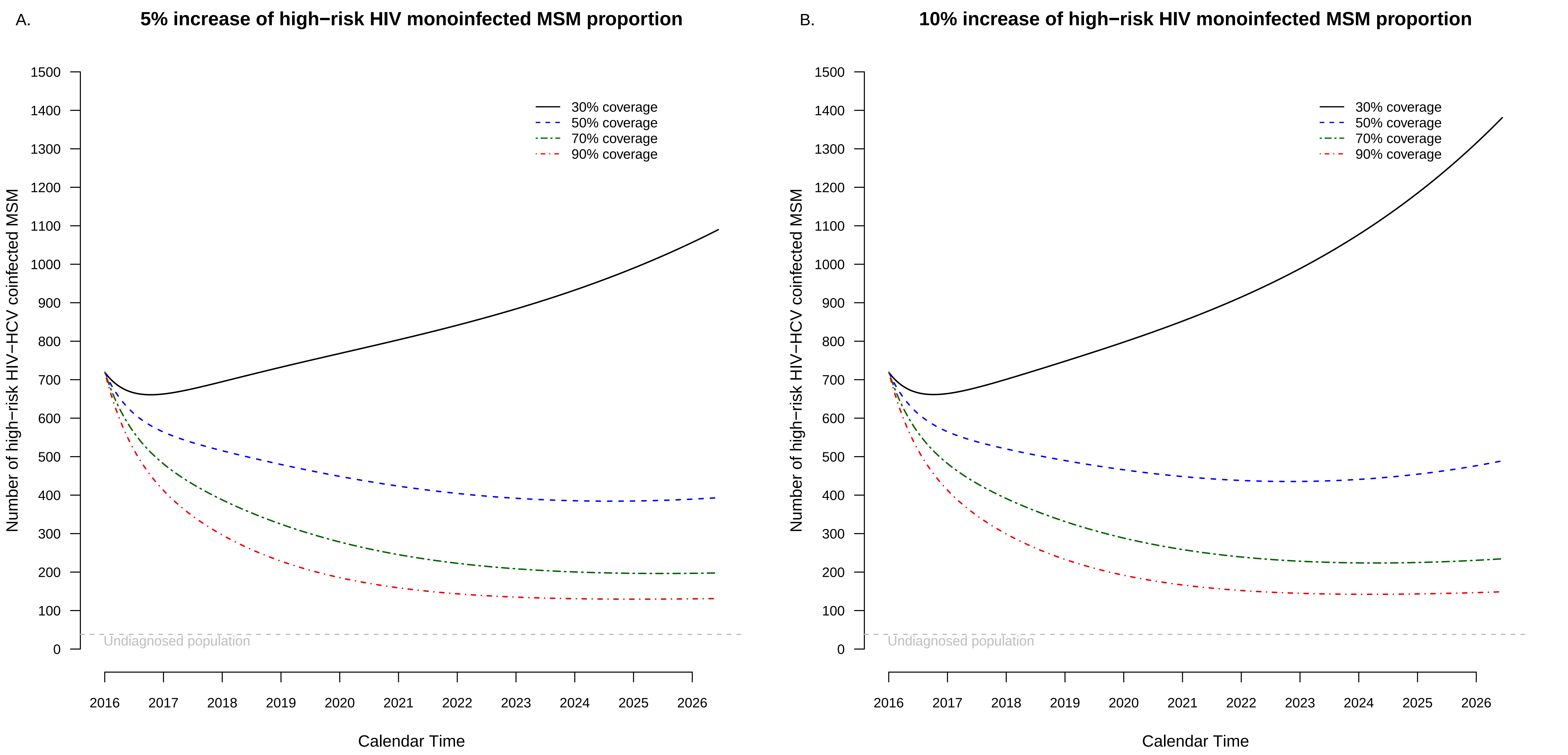
A.
5% increase of high−risk HIV monoinfected MSM proportion
B.
10% increase of high−risk HIV monoinfected MSM proportion
Number of high−risk HIV−HCV coinfected MSM
Calendar Time
30% coverage
50% coverage
70% coverage
90% coverage
Undiagnosed population
0
100
200
300
400
500
600
700
800
900
1000
1100
1200
1300
1400
1500
2016
2017
2018
2019
2020
2021
2022
2023
2024
2025
2026
