# Supplementary material for: Modeling HIV-HCV coinfection epidemiology in the direct-acting antiviral era: the road to elimination
Source: BMC Med. 2017 Dec 18;15:217. doi: 10.1186/s12916-017-0979-1 (PMC5733872; doi:10.1186/s12916-017-0979-1)
Supplement: Supplementary file 8 — Projected prevalence of HIV-HCV coinfections over the next 10 years in IVDU considering a potential risk of HCV transmission between HIV-negative and HIV-positive individuals. (PDF 153 kb) [file 12916_2017_979_MOESM8_ESM.pdf]

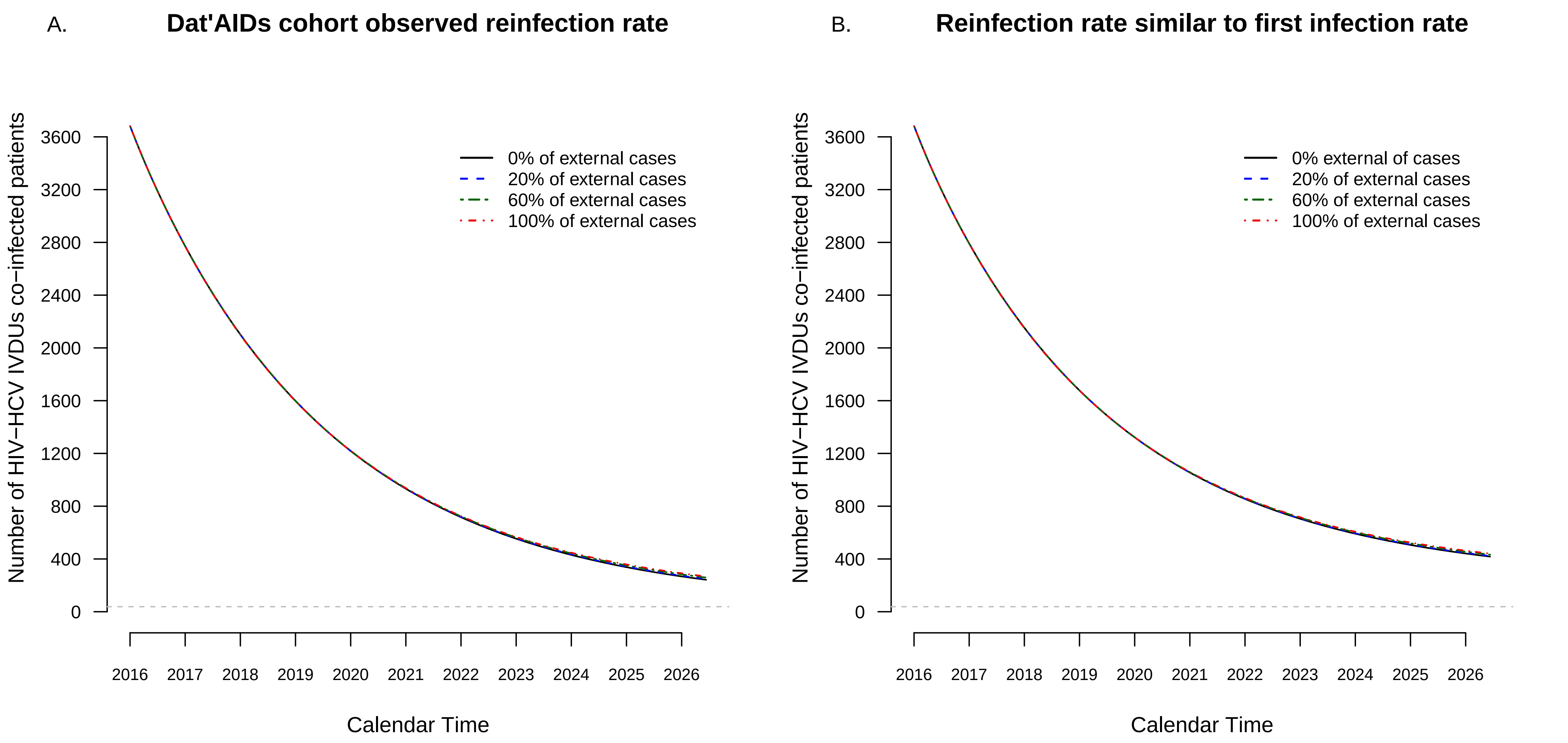

A.
Dat'AIDs cohort observed reinfection rate
B.
Reinfection rate similar to first infection rate
Number of HIV−HCV IVDUs co−infected patients
3600
3200
2800
2400
2000
1600
1200
800
400
0
0% of external cases
20% of external cases
60% of external cases
100% of external cases
0% external of cases
2016
2017
2018
2019
2020
2021
2022
2023
2024
2025
2026
Calendar Time
